# Comparison of laser ablation, simple excision, and flap reconstruction in the treatment of pilonidal sinus disease

**DOI:** 10.1007/s10103-024-03993-5

**Published:** 2024-01-30

**Authors:** Emmi Tyrväinen, Henrik Nuutinen, Elina Savikkomaa, Heidi-Mari Myllykangas

**Affiliations:** 1 Department of Surgery, University of Eastern Finland, Yliopistonranta 8, 70210 Kuopio, Finland; 2 Department of Plastic Surgery, Kuopio University Hospital, PL 1711, 70211 Kuopio, Finland

**Keywords:** Pilonidal sinus, Laser therapy, Surgical flaps, Postoperative complications, Simple excision

## Abstract

Pilonidal sinus disease (PSD) is a common condition that typically affects young adults. PSD may cause significant morbidity due to its chronic nature and tendency to recur. Laser ablation has recently shown promising results in the treatment of PSD, but comparative studies are sparse. We aimed to compare laser ablation with two conventional treatment options: simple excision with direct closure and excision with flap reconstruction. This retrospective study material included patients who underwent PSD surgery in the plastic surgery department of a single academic teaching hospital. Patients were divided into three groups based on the operation technique: laser group, direct closure group, and flap group. Preoperative and postoperative data were compared between the groups including patient characteristics, residual disease, recurrent disease, complications, and re-operations. Among the 278 patients, 66 underwent laser treatment, 134 excision with direct closure, and 78 excision with flap closure. The follow-up time ranged from 15.4 ± 7.6 months in the laser group to 87.6 ± 29.3 months in the flap group. Eventless healing occurred in 67.7% of the patients in the laser group, 66.4% of the patients in the direct closure group, and 56.4% of the patients in the flap group. There was significantly more residual disease in the laser group whereas significantly more complications were found in the direct closure and flap groups. The advantages of laser treatment include fast postoperative recovery and reduced risk of complications.

## Introduction

Sacrococcygeal pilonidal sinus disease (PSD) is an inflammatory condition affecting the subcutaneous tissue of the natal cleft [1]. The incidence of PSD has been reported around 26 per 100.000 [2]. PSD usually occurs in young adults and is more common in males than females [2]. Sinus tracts are formed subcutaneously. They can be asymptomatic or develop painfully acute abscesses as well as chronically discharging secondary openings [1]. Nowadays PSD is considered an acquired disease. Known risk factors include a hairy natal cleft, sedentary work, obesity, sacrococcygeal trauma, and positive family history [2, 3].

There is still a significant morbidity related to PSD despite treatment options. The one significant problem associated with PSD is the tendency to recur after excision [4]. This may lead to repetitive operations and prolonged sick leave. PSD is usually a disease of working age, so in addition to a personal burden, it can cause a significant financial burden for society [5].

Traditional ways to treat PSD include excision with midline closure, off-midline closure with various forms of subcutaneous flaps, as well as excision and healing by secondary intention [6–10]. In recent years, the use of minimal-invasive techniques has increased in the treatment of PSD. These techniques include sinotomy, sinusectomy, and destroying skin pits, sinus tracts, and cavities with phenol or endoscopic ablation [5, 11, 12]. The advantages of minimal-invasive techniques include their less traumatic nature and faster recovery time after the procedure [13–16]. Lately, promising results have been obtained by using laser ablation of the sinus tracts [13–18]. However, only a few studies comparing laser treatment with other techniques have been published [19–21].

Despite many treatment options, the consensus on the optimal care of PSD has yet to be determined [6, 10]. This study aims to compare medium- and long-term results after laser treatment, surgical excision with direct closure, and surgical excision with flap closure.

## Materials and methods

This retrospective study was carried out with a permit of the regional institutional review board (ID 164/2021). Patients, who underwent PSD surgery in the plastic surgery department of a single academic teaching hospital between January 2010 and December 2021 were included in the study. Patients were divided into three groups based on the operation technique: laser group, direct closure group, and flap group. All patients who underwent laser ablation for PSD were included with no exclusion criteria. The direct closure group included all patients operated on with simple excision and simple direct closure of the wound. Patients with any kind of local flap reconstruction were included in the flap group. Patients with whom the wound was left open, either for healing by secondary intention or for closure in a secondary procedure, were excluded from the study.

The need for the surgery was determined at the outpatient clinic. The surgery was recommended for patients with persistent symptoms caused by PSD. The surgical method was chosen based on the severity of the PSD and in accordance with the patient. The laser procedure was introduced in our unit in September 2019, after which it quickly became the first-line treatment of all PSD patients. Before the year 2019, excision with direct closure and multiple types of subcutaneous flap reconstructions were used based on individual assessment. From the multiple flap options, the rotation flap, the rhomboid flap, and the VY flap were the most used.

Procedures were performed in the prone position under local, spinal, or general anesthesia. All patients routinely received a prophylactic dose of cefuroxime (1.5 g) preoperatively. In the laser group, sinus tracts were treated with laser (13 W) at a speed of 1mm/s. The technique has been described earlier [16]. The need for follow-up appointments was determined individually.

The data was collected from the patient records. The following information was collected: age, body mass index (BMI), smoking, diabetes, operation time, anesthesia methods, and the number of fistulas. The collected postoperative data included length of sick leave, duration of the prohibition to sit, postoperative antibiotic use, success of the operation, recurrencies (including residual in 2 months and recurrence after 2 months), postoperative complications (including infection, hematoma, and dehiscence), and the need for re-operation.

The statistical analyses were performed using IBM SPSS Statistics 25.0 software for Windows (SPSS Inc., Chicago, IL, USA). Categorical variables were described as absolute numbers and percentages. Continuous variables were represented by mean with standard deviation. The Mann–Whitney U test was used when comparing two unpaired groups as a non-parametric test. Pearson’s chi-squared test was used when comparing categorical variables. *P*-value < 0.05 was considered statistically significant.

## Results

During the study period, 278 patients were operated; 66 (23.7%) underwent laser treatment, 134 (48.2%) excision with direct closure, and 78 (28.1%) excision with flap closure. The patient groups were comparable concerning age, BMI, diabetes, and the number of fistulas. Smoking was significantly less common in the laser group when compared to the direct closure group (*P* = 0.001) and the flap group (*P* < 0.001). More specific data is presented in Table 1.

**Table 1:** The characteristics of the patient groups

	Laser (66)	Direct closure (134)	Flap (78)
Patients, *n* (%)	66 (23.7)	134 (48.2)	78 (28.1)
DM1, *n* (%)	1 (1.5)	3 (2.2)	3 (3.8)
DM2, *n* (%)	1 (1.5)	2 (1.5)	2 (2.6)
Smoking, *n* (%)	16 (24.2)	63 (47.0)	35 (55.9)
Age, mean ± SD	27.8 ± 9.9	28.1 ± 10.3	30.4 ± 11.5
BMI, mean ± SD	27.4 ± 4.8	29.1 ± 6.1	30.5 ± 11.5
No of fistulas, mean ± SD	3.0 ± 1.6	1.9 ± 1.6	2.5 ± 1.5
Follow-up, mean ± SD	15.4 ± 7.6	85.2 ± 39.8	87.6 ± 29.3

*DM1* Diabetes mellitus, type 1, *DM2* Diabetes mellitus, type II, *BMI* body mass index

Most of the patients were treated with day surgery. Spinal anesthesia was the commonly used type of anesthesia in the laser group and the direct closure group, whereas general anesthesia was the most common type of anesthesia in the flap group (Table 2). The mean operative time was significantly shorter in the laser group (19.5 min) than in the direct closure group (35.6 min) or the flap group (57.3 min). Additionally, the mean length of sick leave (in days) was shorter in the laser group (6.2 ± 8.9) compared to the direct closure group (27.4 ± 27.0) or the flap group (29.6 ± 14.5). The need for prohibition to sit (in weeks) was significantly shorter after laser treatment (0.0 ± 0.4) than after direct closure (2.5 ± 1.1) or flap reconstruction (3.2 ± 1.1). All these differences were statistically significant (*P*-values < 0.001).

**Table 2:** Intra- and postoperative data

	Laser (66)	Direct closure (134)	Flap (78)
Operation time, (in min), mean ± SD	19.5 ± 7.3	35.6 ± 17.8	57.3 ± 16.7
Day surgery, *n* (%)	62 (93.9)	94 (70.1)	53 (67.9)
Local anesthesia, *n* (%)	8 (12.1)	4 (3.0)	1 (1.3)
Spinal anesthesia, *n* (%)	49 (74.2)	65 (48.5)	23 (29.5)
General anesthesia, *n* (%)	9 (13.6.)	60 (44.8)	54 (69.2)
Anesthesia – missing information	0	5 (3.7)	0
Postop antibiotics, *n* (%)	3 (4.5)	62 (46.3)	68 (87.2)
Prohibition to sit (in weeks), mean ± SD	0.0 ± 0.4	2.5 ± 1.1	3.2 ± 1.1
Sick leave (in days), mean ± SD	6.2 ± 8.9	27.4 ± 27.0	29.6 ± 14.5
No of controls, mean ± SD	3.4 ± 3.9	1.8 ± 2.6	1.6 ± 3.0

The follow-up time was 15.4 ± 7.6 months in the laser group and significantly longer in the direct closure and flap groups (85.2 ± 39.8 months, 87.6 ± 29.3 months, respectively).

Eventless healing without any residual disease, recurrence, or complication was accomplished in 44 (67.7%) patients in the laser group, 89 (66.4%) patients in the direct closure group, and 44 (56.4%) patients in the flap group. Residual disease within two months after surgery was most common in the laser group. This difference was statistically significant when comparing the laser group (25.8%) separately to the direct closure group (8.2%, *P* = 0.001) and to the flap group (7.7%, *P* = 0.003). However, there were significantly more complications in the direct closure group (26.9%, *P* = 0.018) and in the flap group (34.6%, *P* = 0.003) when compared to the laser group (12.1%). There were no statistically significant differences in re-operation rates between the groups. More detailed results of recovery are presented in Table 3.

**Table 3:** Residual disease, recurrent disease, and complications

	Laser (66)	Direct closure (134)	Flap (78)
Eventless healing*, *n* (%)	44 (67.7)	89 (66.4)	44 (56.4)
Residual in 2 months, *n* (%)	17 (25.8)	11 (8.2)	6 (7.7)
Recurrence after 2 months, *n* (%)	2 (3.1)	10 (7.5)	9 (11.7)
Any complication^, *n* (%)	8 (12.1)	36 (26.9)	27 (34.6)
Infection, *n* (%)	8 (12.1)	18 (13.4)	17 (21.8)
Hematoma, *n* (%)	0	7 (5.2)	6 (7.7)
Dehiscence, *n* (%)	0	27 (20.1)	11 (14.1)
New operation, *n* (%)	13 (19.7)	21 (15.7)	12 (15.4)

*No residual disease, recurrence, or complications, ^Same patient may have several complications

## Discussion

Multiple options have been described in the treatment of pilonidal disease from mini-invasive treatments to more extensive flap reconstructions [4, 8, 10]. The recurrence rates have been reported as high as 67.9% during a long 240-month follow-up [10].

In a search for a more optimal treatment for the pilonidal disease, laser treatment has recently become a valuable option in many centers [18]. Despite numerous patient series published concerning laser treatment, comparative studies are sparse. Abdelnaby et al. compared laser treatment to the open lay technique. In their study, they reported faster healing and better quality of life in the laser treatment group [21]. Yerdimci et al. compared laser treatment with flap reconstruction in a randomized study with 30 and 28 patients in each group. They reported shorter operation time, less pain, and better patient satisfaction with laser treatment [20]. Similar results have been published in a prospective non-randomized study by Algazar et al. [19].

The systematic review by Romic et al. reported a recurrence rate as low as 3.8% after laser treatment [18]. However, the median follow-up time of 12 months may be considered limited, as the length of follow-up time has been considered a major factor affecting the results in many studies [4, 10]. The superiority of laser treatment over other treatment options considering the recurrence rate or late results has not been proved in any comparative studies.

In our results, the rate of residual disease was slightly higher when compared to earlier studies [18]. However, our complication rates with laser treatment were lower when compared to open surgery. This resulted in a comparable number of patients with eventless healing. There were slightly fewer patients with eventless healing in the flap group. This was most likely explained by the fact that the flap group contained more difficult cases than the two other groups. Patients’ characteristics such as age, BMI, diabetes, and smoking may affect the healing process. Smoking was significantly less common in the laser groups when compared to the other groups. This is probably due to the decreasing rate of smoking and may favor the patients in the laser groups in these comparisons.

In our hospital, the first patient was operated with laser treatment in September 2019. After January 2020, only four patients were operated with open surgery. This means that laser treatment replaced both excision with direct closure and excision with a flap reconstruction as treatment options. Before 2020, patients were selected either for direct closure or flap reconstruction based on individual decision-making in the outpatient clinic. Most often the patients with more difficult disease presentation, and many fistulas affecting a large area of the skin, were selected for the flap reconstruction. However, this kind of selection bias should not affect the comparisons between the patients in the laser group and the other two groups, since the patients were operated mostly during different periods. However, because the patients in the different groups were operated during different periods, the follow-up times are not comparable.

The patients presented here in the laser group represent the very first patients operated on with this technique in our hospital. Later, the treatment was carried out with local anesthesia in the outpatient clinic settings with rare exceptions. The length of sick leave and operation time has also gotten shorter over time. In addition, the more numerous controls in the laser treatment group represent the extra controls associated with the initiation of the new technique. Unfortunately, the follow-up schedule was not standard due to the retrospective nature of this study.

Our study is limited by its retrospective nature and confounding factors in patient selection between the direct closure and the flap reconstruction groups. In the future, randomized studies with long follow-up periods are warranted to prove the efficacy of laser treatment in pilonidal disease.

Laser treatment may not be the perfect solution for the treatment of this disease with a high potential to relapse. However, open surgery and flap reconstruction are associated with the risk of major complications, laborious post-operative care, long prohibition to sit, and lengthy sick leave. These factors are associated with direct and indirect costs and influence the patient´s quality of life during the postoperative phase. Consequently, it seems reasonable to consider laser treatment, and even a second one, before progressing to the more invasive surgical options.

## Conclusion

The advantages of laser treatment are fast postoperative recovery and reduced risk of complications. Despite the higher risk of recurrence compared to surgical excisions, laser treatment should be considered as a primary treatment option before resorting to more invasive procedures.
